# PA-X antagonises MAVS-dependent accumulation of early type I interferon messenger RNAs during influenza A virus infection

**DOI:** 10.1038/s41598-019-43632-6

**Published:** 2019-05-10

**Authors:** Rachel E. Rigby, Helen M. Wise, Nikki Smith, Paul Digard, Jan Rehwinkel

**Affiliations:** 1 Medical Research Council Human Immunology Unit, Medical Research Council Weatherall Institute of Molecular Medicine, Radcliffe Department of Medicine, University of Oxford, Oxford, UK; 2 The Roslin Institute & Royal (Dick) School of Veterinary Studies, University of Edinburgh, Edinburgh, UK; 3 Present Address: Clinical biochemistry, Clock Tower building, Western General Hospital, Edinburgh, UK

**Keywords:** RIG-I-like receptors, Influenza virus

## Abstract

The sensing of viral nucleic acids by the innate immune system activates a potent antiviral response in the infected cell, a key component of which is the expression of genes encoding type I interferons (IFNs). Many viruses counteract this response by blocking the activation of host nucleic acid sensors. The evolutionarily conserved influenza A virus (IAV) protein PA-X has been implicated in suppressing the host response to infection, including the expression of type I IFNs. Here, we characterise this further using a PA-X-deficient virus of the mouse-adapted PR8 strain to study activation of the innate immune response in a mouse model of the early response to viral infection. We show that levels of *Ifna4* and *Ifnb1* mRNAs in the lungs of infected mice were elevated in the absence of PA-X and that this was completely dependent on MAVS. This therefore suggests a role for PA-X in preventing the accumulation of early type I IFN mRNAs in the lung during IAV infection.

## Introduction

Influenza A virus (IAV) is a major cause of human respiratory infections with a disease pathogenesis that varies widely depending on the strain of virus. Viral infection activates the host innate immune response which the virus must circumvent in order to establish a productive infection. Detection of viral genomes by the cytosolic RNA helicase Retinoic acid-Inducible Gene I (RIG-I) is a key trigger for type I interferon (IFN) production during IAV infection^1,2^. Upon binding the viral RNA, RIG-I undergoes a conformational transformation which allows it to interact with Mitochondrial Antiviral Signalling protein (MAVS), a signalling adaptor protein located at the outer mitochondrial membrane. This triggers oligomerisation of MAVS and subsequent recruitment of signalling molecules which activate the transcription factors IFN Regulatory Factor (IRF) 3 and IRF7 which stimulate expression of genes encoding type I and type III interferons. Consequently, several IAV proteins act to antagonise the RIG-I-MAVS pathway, including NS1^3^ and PB1-F2^4^.

The genome of IAV consists of eight negative sense single-stranded RNA segments. In addition to PA, which is an essential component of the viral RNA polymerase complex, segment 3 of IAV also encodes the accessory protein PA-X which is generated as a consequence of ribosomal frame-shifting^5^. During translation, the ribosome can stall at a rare codon within the PA open reading frame (ORF), which occasionally results in shifting to a +1 ORF, termed the ‘X-ORF’. This results in the production of a truncated protein, PA-X, which consists of the 191 amino acid (aa) N-terminal endoribonuclease domain of PA fused to a novel C-terminal domain. The frameshift sequence in segment 3 is highly conserved, suggesting that PA-X can be produced by all IAV strains although the novel C-terminal domain can commonly be either 61 aa or 41 aa, depending on viral isolate^6^.

Experimental infection of mice with IAV lacking PA-X has indicated a role for it in the suppression of the host immune response to IAV infection^7,8^. PA-X-deficient viruses of the 1918 H1N1, 2009 pH1N1 and H5N1 strains induced increased pro-inflammatory cytokines and type I IFNs in the lungs compared to infection with wild-type viruses that expressed PA-X^5,9–12^. We therefore investigated whether PA-X has a role in modulating the host response to IAV infection by preventing activation of the RIG-I pathway using a PA-X deficient virus of the highly mouse-adapted A/Puerto Rico/8/1934 H1N1 (‘PR8’) strain. We show that this PA-X-deficient PR8 virus induced increased expression of *Ifnb1* and *Ifna4* mRNAs in the lungs of infected mice compared to a PA-X-expressing PR8 virus. Surprisingly, however, this did not correlate with increased type I IFN protein or expression of interferon stimulated genes (ISGs). Furthermore, we show that increased *Ifnb1* and *Ifna4* mRNA levels induced by PA-X-deficiency were completely dependent on MAVS. We therefore conclude that the evolutionarily conserved viral protein PA-X can act to specifically prevent accumulation of early type I IFN mRNAs via a MAVS-dependent pathway during IAV infection.

## Results

### Generation of a PA-X deficient PR8 virus

**Figure 1:**
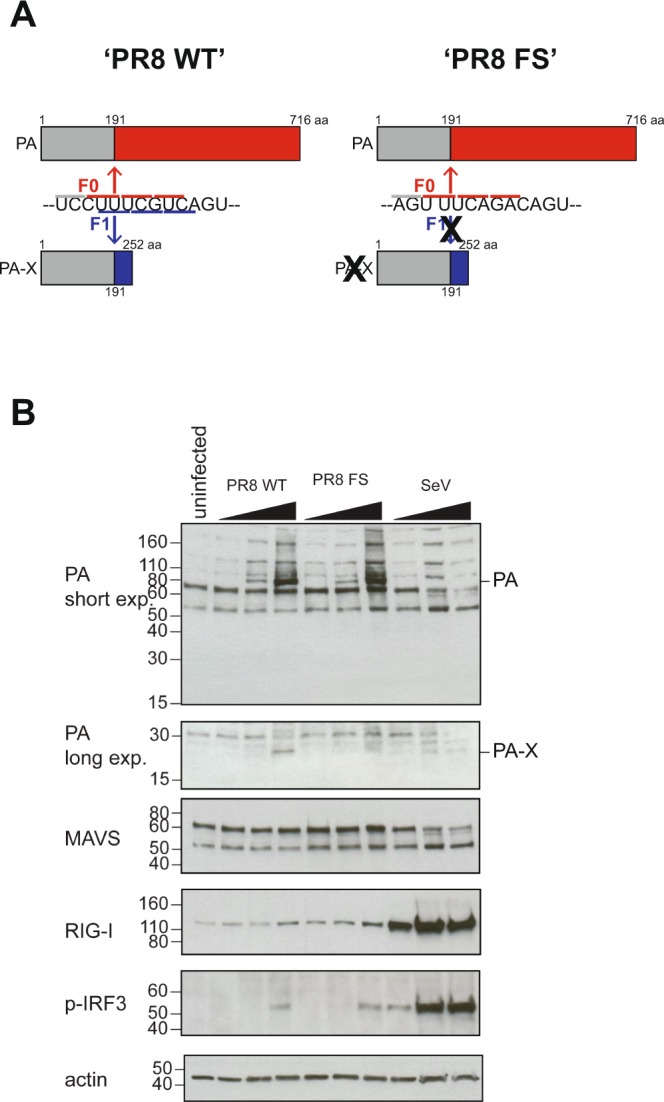
Generation of a PA-X-deficient PR8 virus. (**A**) Schematic illustration of the two viruses used in this study: ‘PR8 WT’ and ‘PR8 FS’ which has greatly reduced translation of PA-X due to mutations in the frameshift motif. (**B**) HEK293T cells, pre-treated overnight with 100 U/ml IFN-A/D to increase expression of RIG-I to detectable levels, were infected with PR8 WT, PR8 FS or Sendai virus (SeV) at increasing MOIs (0.1, 1, 10). Cells were harvested for immunoblotting 24 h.p.i. using the indicated antibodies. Full-length blots are shown in Fig. S2.

To investigate the contribution of PA-X to the innate immune response to IAV in an *in vivo* model, we used reverse genetics to generate wild-type and PA-X-deficient PR8 viruses^13^. PR8 is a mouse-adapted strain of IAV which is commonly used to model IAV infection in susceptible inbred mouse strains, including C57BL/6^14^. The PR8 PA-X protein shows relatively low-shutoff activity compared to PA-X proteins from H5N1 and H7N1 IAV strains when tested *in vitro* in cells^13^. The PA-X-deficient virus (‘PR8 FS’) contains mutations in the ribosomal frameshifting motif (UCC UUU CGU to AGU UUC AGA) (Fig. 1A) which reduces the frameshifting efficiency to less than 0.1% without affecting the expression of the full-length PA segment^5^. There was no difference in replication rates between PR8 WT and PR8 FS in A549 cells (Fig. S1). We then infected human HEK293T cells with the two different viruses and confirmed the full-length viral PA protein was detectable at equivalent levels but PA-X was present only in cells infected with the parental PR8 virus using a polyclonal antibody raised against the N-terminus of the PA protein (Fig. 1B; full-length blots are shown in Fig. S2). Both viruses were able to induce innate immune signalling in this human cell line, as shown by phosphorylation of IRF3 and increased levels of RIG-I which is encoded by the ISG *DDX58* (Fig. 1B) compared to mock infected cells. In these experiments, Sendai virus (Cantell strain), a strong inducer of type IFNs, served as a positive control.

### *In vivo* infection with a PA-X deficient PR8 virus increases expression of *Ifnb1* and *Ifna4* mRNA in the lung

**Figure 2:**
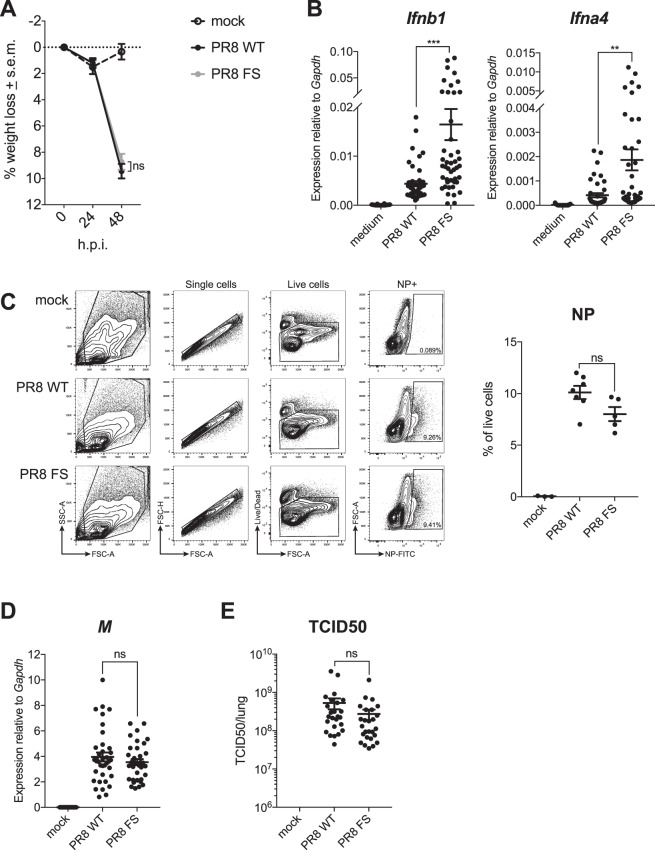
PA-X reduces expression of *Ifnb1* and *Ifna4* mRNA in the lungs of infected mice. Wild-type mice were infected with 50,000 pfu of PR8 WT, PR8 FS or mock infected via the intranasal route. Mice were weighed every 24 h and sacrificed 48 h.p.i. (**A**) Weight loss in mice infected with PR8 WT and PR8 FS. Data are represented as mean weight loss from 13 (mock), 40 (PR8 WT) and 39 (PR8 FS) mice per group ± s.e.m., pooled from 11 independent experiments using two independently prepared virus stocks. (**B**) Levels of *Ifnb1* and *Ifna4* mRNA in the lung were measured by qRT-PCR. Data are from 26 (mock) and 40–47 (infected) mice per group and are represented as expression relative to the housekeeping gene *Gapdh* ± s.e.m., pooled from 15 independent experiments. (**C**) The proportion of NP+ cells in the lung was quantified by flow cytometry. Exemplary FACS plots are shown on the left. Data are represented as percentage of single, live cells ± s.e.m and are from 3 (mock), 7 (PR8 WT) and 5 (PR8 FS) mice per group, pooled from two independent experiments (right). (**D**) Levels of the IAV M RNA were measured by qRT-PCR. Data are from 17 (mock), 37 (PR8 WT) and 35 (PR8 FS) mice per group ± s.e.m. and are pooled from nine independent experiments. (**E**) Viral loads in the right lungs of infected mice were determined using a TCID_50_ assay. Data are from 16 (mock; none detected) and 26 (infected) mice per group ± s.e.m. and are pooled from six independent experiments. *P ≤ 0.05, **P ≤ 0.01, ***P ≤ 0.001, ns not significant; unpaired Student’s *t-*test.

To investigate the effect of PA-X-deficiency on the innate immune response to PR8 *in vivo*, we infected C57BL/6 mice with 50,000 pfu of each virus via the intranasal route and monitored animals for up to 72 h.p.i. (Fig. S3). We chose this dose and the 48 h.p.i. timepoint for subsequent experiments because this was when levels of both *Ifnb1* and *Ifna4* mRNA were at their highest following infection without causing overt suffering to the animal, allowing us to investigate the initial innate immune response. As expected, IAV-infected mice lost a proportion of their body weight during the course of the infection; however, there was no significant difference in weight loss between PR8 WT and PR8 FS-infected mice at 48 h.p.i. (8.7% versus 9.4% by 48 h.p.i.) (Fig. 2A), indicating that both viruses caused a similar degree of morbidity.

Recognition of viral RNA by the pattern recognition receptors (PRRs) of the innate immune system activates signalling pathways which induce the transcription of genes encoding cytokines, most notably type I interferons (IFNs). To investigate whether the presence or absence of PA-X has an effect on the induction of type I IFN mRNA, we performed qRT-PCR on total RNA extracted from the lungs of infected mice 48 h.p.i. As expected, IAV infection induced both *Ifnb1* and *Ifna4* expression in the lungs compared to mock-infected animals; however, this was significantly elevated in the lungs of mice infected with the PA-X-deficient PR8 virus compared to wild-type PR8 (Fig. 2B). This was observed in multiple independent experiments, the data for which are shown pooled in Fig. 2B. Increased levels of *Ifnb1* mRNA in the lungs of PR8 FS-infected mice could also be detected by Northern blot (Fig. S4). To exclude the possibility that this was due to enhanced replication of the PA-X-deficient virus, we identified infected cells using flow cytometry. We observed equivalent numbers of lung cells positive for the IAV NP protein in samples from animals infected with either virus (Fig. 2C). We also quantified the levels of the viral *M* RNA segment by qRT-PCR in the lungs of infected mice and found no difference between the two viruses (Fig. 2D). To compare the amount of actively replicating virus in the lungs of infected mice, we measured viral titres and found no difference between PR8 WT and PR8 FS-infected mice (Fig. 2E). Taken together, these data show that the PR8 WT and PR8 FS viruses are equally virulent but *Ifnb1* and *Ifna4* mRNA levels are elevated in the lungs of mice infected with the PA-X-deficient virus.

### Levels of type I IFN protein and expression levels of IFN-dependent genes are not elevated in the absence of PA-X

**Figure 3:**
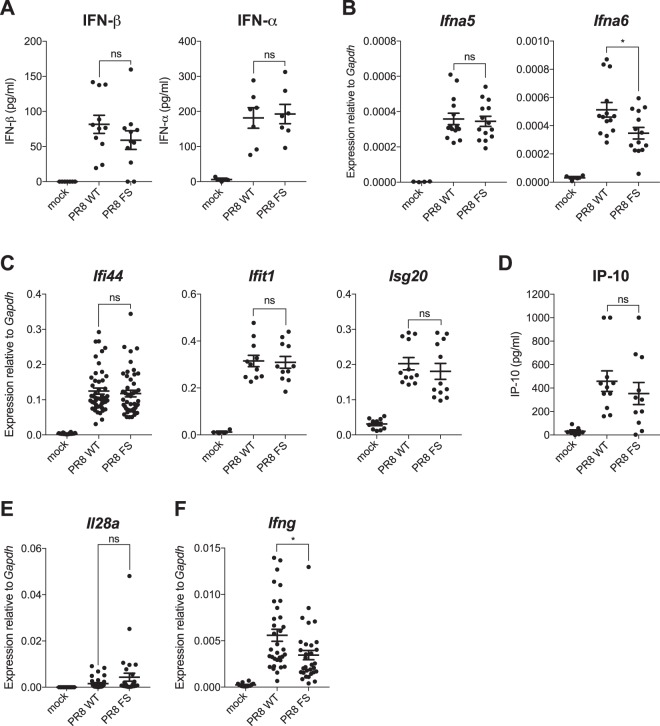
PA-X does not affect the expression of type I IFN protein or ISGs in the lung. Wild-type mice were infected with 50,000 pfu of PR8 WT, PR8 FS or mock infected via the intranasal route and sacrificed 48 h.p.i. (**A**) Quantification of IFN-β and IFN-α protein in the BAL fluid by ELISA. Data are from 3–7 (mock) and 7–12 (infected) mice per group ± s.e.m. and are pooled from four independent experiments. (**B**) Levels of *Ifna5* and *Ifna6* mRNA in the lung were measured by qRT-PCR. Data are from 4 (mock) and 14 (infected) mice per group and are represented as expression relative to the housekeeping gene *Gapdh* ± s.e.m., pooled from 4 independent experiments. (**C**) *Ifi44*, *Ifit1* and *Isg20* mRNA levels in the lungs were quantified using qRT-PCR. Data are from 4–28 (mock) and 11–49 (infected) mice per group ± s.e.m. and are pooled from 11 (*Ifi44*) or three (*Isg20* and *Ifit1*) independent experiments. (**D**) Quantification of IP-10 protein in the BAL fluid by ELISA. Data are from 7 (mock) and 11 (infected) mice per group ± s.e.m. and are pooled from four independent experiments. Expression of (**E**) *Il28a* and (**F**) *Ifng* in the lungs were quantified by qRT-PCR. Data are from 14 (mock) and 30–31 (infected) mice per group ± s.e.m. and are pooled from 8 independent experiments. *P ≤ 0.05, ns not significant; unpaired Student’s *t-*test.

We expected that cells expressing elevated levels of *Ifnb1* and *Ifna4* mRNA secrete more IFN-β and IFN-α protein. To confirm this, we quantified the amount of IFN-β and IFN-α in the broncheoalveolar lavage fluid (BALf) of mice infected with either PR8 WT or PR8 FS and found that, surprisingly, there was no significant difference in the concentration of these type I IFNs in the lungs of the two infected groups (Fig. 3A).

*Ifnb1* and *Ifna4* are unusual amongst type I IFN genes as their expression is induced early in response to infection in an IFN protein-independent manner, although this may be cell type dependent^15–17^. In contrast, the other members of the *Ifna* gene family require a positive feedback loop involving synthesis of IFN protein for their expression and therefore exhibit delayed kinetics of induction following viral infection. We quantified the mRNA levels of two different *Ifna* family members, *Ifna5* and *Ifna6*, in the lungs of infected mice. Expression of both genes was induced in response to infection with IAV; however, expression levels were comparable between PR8 WT and PR8 FS-infected mice, or slightly reduced in PR8 FS-infected mice (Fig. 3B), suggesting that PA-X deficiency does not increase the expression of all type I IFN genes in the lungs.

A key process in shaping an antiviral response is the binding of secreted type I IFN proteins to the type I interferon receptor (IFNAR) which is expressed on the surface of all cell types. This leads to the triggering of multiple downstream signalling events and to the transcriptional activation of hundreds of ISGs. These encode a variety of proteins which can act to enhance both the innate and adaptive immune responses to viral infection^18^. We measured the mRNA levels of three different ISGs (*Ifi44*, *Ifit1* and *Isg20*) in the lungs of infected mice using qRT-PCR. Transcription of all of these ISGs was induced in response to IAV infection, and PR8 FS-infected lungs had equivalent expression to those infected with PR8 WT (Fig. 3C). Similarly, there was no difference in the concentration of the IFN-inducible chemokine IP-10 (CXCL10) in BALf (Fig. 3D). Together, these data show that the increased levels of *Ifnb1* and *Ifna4* mRNA in the absence of PA-X do not correlate with an increase in the downstream effects of IFN-dependent signalling, possibly due to there being no concurrent increase in the level of IFN-β and IFN-α protein.

The failure of mRNA to be translated into protein can occur if the 3′-end of the mRNA has not been correctly polyadenylated. To investigate if the *Ifnb1* mRNA from the lungs of PR8 FS-infected mice was polyadenylated, we analysed the same RNA samples by qRT-PCR using random hexamers or oligo(dT) oligonucleotides to generate the cDNA. Increased *Ifnb1* levels in RNA samples from FS-infected mice were detected when oligo(dT) oligonucleotides were used to prime the reverse transcription reaction (Fig. S5A), indicating that *Ifnb1* mRNA from the lungs of FS-infected mice is polyadenylated. As poly(A) tail length is known to affect the ability of mRNA transcripts to be translated we used extension poly(A) test (ePAT)^19^ to measure the length of the *Ifnb1* mRNA poly(A) tail. This showed that *Ifnb1* mRNA extracted from the lungs of mice infected with PR8 FS had a full poly(A) tail, identical in length to that of *Ifnb1* mRNA from the lungs of mice infected with the WT virus (Fig. S5B). Therefore, *Ifnb1* mRNA transcribed in the presence or absence of PA-X was correctly polyadenylated.

Finally, we investigated the effect of PA-X-deficiency on the expression of type II and type III IFN genes. Members of the type III IFN (IFN-λ) family are distantly related to type I IFNs and share some overlapping antiviral functions^20^. Expression of the IFN-λ-encoding genes in the lungs is a key part of the antiviral response to IAV^21^. To investigate if PA-X-deficiency also affects the expression of type III IFNs, we quantified expression of *Il28a* (which encodes IFN-λ2) by qRT-PCR. There was no significant increase in the expression of *Il28a* in the lungs of PR8 FS-infected mice compared to PR8 WT-infected mice (Fig. 3E). Likewise, PA-X-deficiency did not result in increased expression of *Ifng* which encodes the only member of the type II IFN family and is also induced in response to infection with IAV (Fig. 3F).

### The lungs of PR8 FS-infected mice contain less immunostimulatory RNA

**Figure 4:**
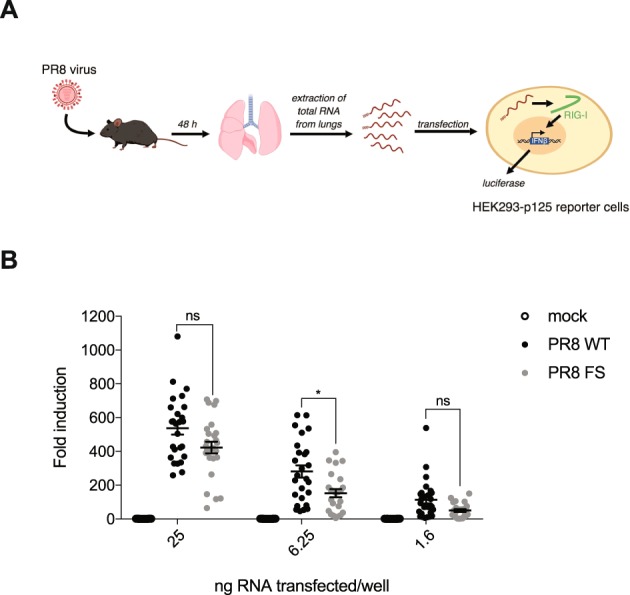
The lungs of mice infected with PA-X deficient virus contain less immunostimulatory RNA. (**A**) Schematic of the assay using HEK293-p125 reporter cells. (**B**) RNA extracted from the lungs of infected mice was transfected into HEK293-p125 cells. 18 hours later, induction of the *IFNB* promoter was determined. Data are shown as fold induction compared to cells treated with transfection reagent only and are from 20 (mock), 26 (PR8 WT) and 24 (PR8 FS) mice per group ± s.e.m. and are pooled from five independent experiments. *P ≤ 0.05, ns not significant; two-way ANOVA.

Viral RNA is a potent activator of the innate immune response. Total RNA extracted from IAV-infected cells using an organic extraction reagent, such as TRIzol, which strips viral proteins leaving only naked viral RNA, strongly induces IFN-β production via RIG-I when transfected into uninfected cells^1,22^. To investigate whether the increased expression of *Ifnb1* and *Ifna4* mRNA in the lungs of PR8 FS-infected mice was due to an accumulation of immunostimulatory viral RNA which would otherwise be degraded by PA-X, we transfected RNA isolated from the lungs of mice infected with PR8 WT or PR8 FS or mock infected into HEK293-p125 reporter cells, which stably express firefly luciferase under the control of the human *IFNB* promoter^23^ (Fig. 4A). Unexpectedly, at an intermediate, non-saturating dose, there was less *IFNB*-inducing RNA in the lungs of PR8 FS-infected mice than in the lungs of PR8 WT-infected mice (Fig. 4B). Therefore, the increased expression of the *Ifnb1* and *Ifna4* genes is unlikely to be due to increased amounts of immunostimulatory RNA in the cells of PR8 FS-infected lungs.

### PA-X reduces activation of a MAVS-dependent pathway

IAV genomic RNA is predominantly detected via two RNA sensing pathways of the innate immune system which recognise it as pathogenic. Viral RNA released from virions entering the endosomal compartment activates TLR7^24^. Detection of replicating viral RNA is via the binding of 5′-triphosphate containing viral genomes to the RNA helicase RIG-I in the cytosol of infected cells^1,2^. Following the binding of viral RNA, RIG-I undergoes conformational changes which allow binding to the signaling adaptor MAVS, resulting in the production of type I IFNs and pro-inflammatory cytokines^25^.

**Figure 5:**
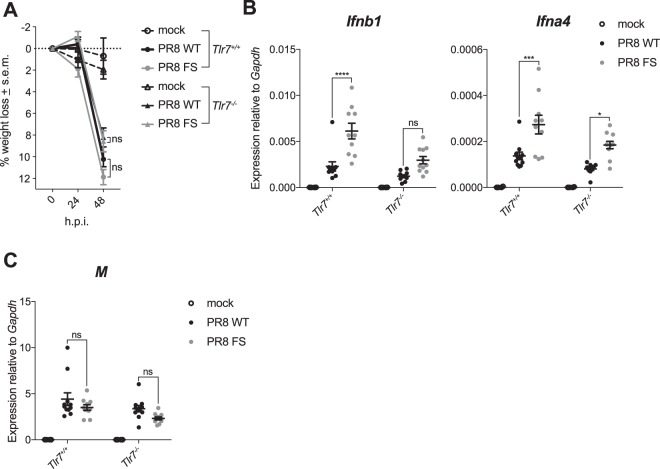
PA-X reduces activation of a TLR7-independent pathway. Wild-type and *Tlr7*^−/−^ mice were infected with 50,000 pfu of PR8 WT, PR8 FS or mock infected via the intranasal route. Mice were weighed every 24 h and sacrificed 48 h.p.i. (**A**) Weight loss in mice infected with PR8 WT and PR8 FS. Data are represented as mean weight loss ± s.e.m. (**B**) Levels of *Ifnb1* and *Ifna4* mRNA in the lung were measured by qRT-PCR. (**C**) Levels of the IAV M RNA were measured by qRT-PCR. Data are represented as expression relative to the housekeeping gene *Gapdh* ± s.e.m. Data in (**A–C**) are from 4 (mock) and 10–11 (infected) mice per group mice per group, pooled from three independent experiments. *P ≤ 0.05, ***P ≤ 0.001, ns not significant; two-way ANOVA.

To investigate via which RNA sensing pathway the increased type I IFN mRNA is induced in the lungs of mice infected with the PA-X-deficient PR8 virus, we first infected mice lacking TLR7 with PR8 WT and PR8 FS. Similar to TLR7-sufficient mice, there was no difference in weight loss in *Tlr7*^−/−^ mice infected with the different viruses (Fig. 5A). Levels of *Ifnb1* and *Ifna4* mRNA were lower overall in PR8-infected *Tlr7*^−/−^ mice compared to wild-type mice, reflecting the contribution of TLR7 to type I IFN production in IAV infection. However, expression of both type I IFNs was higher in PR8 FS-infected mice compared to PR8 WT-infected mice (Fig. 5B) with no difference in the expression of IAV *M* RNA (Fig. 5C). Therefore, PA-X is affecting type I IFN mRNA expression via a TLR7-independent mechanism.

**Figure 6:**
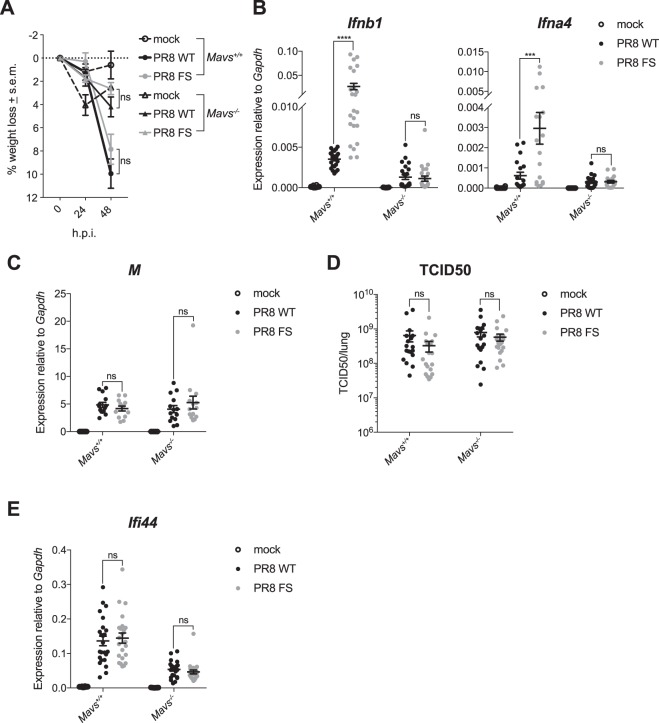
PA-X reduces activation of a MAVS-dependent pathway. Wild-type and *Mavs*^−/−^ mice were infected with 50,000 pfu of PR8 WT, PR8 FS or mock infected via the intranasal route. Mice were weighed every 24 h and sacrificed 48 h.p.i. (**A**) Weight loss in mice infected with PR8 WT and PR8 FS. Data are represented as mean weight loss ± s.e.m and are from 3 (mock) and 9–10 (infected) mice per group, pooled from two independent experiments. (**B**) Levels of *Ifnb1* and *Ifna4* mRNA in the lung were measured by qRT-PCR. Data are represented as expression relative to the housekeeping gene *Gapdh* ± s.e.m. and are from 11–16 (mock) and 18–24 (infected) mice per group and are pooled from five independent experiments. (**C**) Expression of the IAV M RNA was measured by qRT-PCR. Data are from 7 (mock) and 13–14 (infected) mice per group ± s.e.m and are pooled from three independent experiments. (**D**) Viral loads in the right lungs of infected mice were determined using a TCID_50_ assay. Data are from 14 (mock; none detected) and 18–19 (infected) mice per group ± s.e.m and are pooled from four independent experiments. (**E**) Expression of *Ifi44* mRNA in the lungs was quantified by qRT-PCR. Data are from 9–16 (mock) and 23–24 (infected) mice per group ± s.e.m and are pooled from five independent experiments. ***P ≤ 0.001, ****P ≤ 0.0001, ns not significant; unpaired Student’s *t-*test (**A**) and two-way ANOVA (**B–E**).

We next investigated whether PA-X was affecting activation of a RIG-I-dependent pathway by infecting mice lacking the adaptor protein MAVS, as RIG-I knockout mice are embryonic lethal on the C57BL/6 background^26^. Surprisingly, *Mavs*^−/−^ mice lost less weight than wild-type control mice upon IAV infection, which should be investigated in future studies. However, there was no difference in weight loss between *Mavs*^−/−^ mice infected with PR8 WT or PR8 FS, mirroring the situation in wild-type animals (Fig. 6A). Interestingly, in contrast to wild-type mice, there was no increase in the expression of *Ifnb1* and *Ifna4* in the lungs of *Mavs*^−/−^ mice infected with PR8 FS compared to *Mavs*^−/−^ mice infected with PR8 WT (Fig. 6B). Similar to wild-type mice, there was no difference in the expression of viral RNA (Fig. 6C), viral load (Fig. 6D) or the expression of ISGs (Fig. 6E) in the lungs of *Mavs*^−/−^ mice infected with either PR8 WT or PR8 FS.

Taken together, our data show that PA-X inhibited the expression of *Ifnb1* and *Ifna4* by a MAVS-dependent pathway, most likely via activation of RIG-I.

## Discussion

IAV, like many other viruses, has evolved mechanisms to counteract and evade the immune response that is activated when an infected cell is alerted to the presence of a pathogen. The production of type I IFNs is a key aspect of the innate immune response to virus infection, driving the induction of a multitude of ISGs which help to establish an antiviral state in an infected tissue. The multifunctional IAV protein NS1 is a potent antagonist of the innate immune response. It has a number of different viral strain-specific modes of action which include inhibiting mRNA processing, translation and inflammasome activation (reviewed by Krug^3^). In particular, NS1 has a number of different mechanisms to specifically target and block IFN induction via the RIG-I-MAVS pathway. These include sequestration of immunostimulatory RNAs^1,27^, binding to *Ddx58* pre-mRNA^28^, directly interacting with the RIG-I^27,29,30^ and TRIM25 proteins^31^ and the inhibition of the transcription factors IRF3^32^ and NF-κB^33^. The RIG-I-MAVS pathway is also targeted by other IAV proteins, including PB2 and PB1-F2, which bind to MAVS^4,34,35^. Here, we showed that a another IAV protein, PA-X, prevents activation of the RIG-I-MAVS pathway, resulting in reduced levels of *Ifnb1* and *Ifna4* mRNAs.

Both NS1 and PA-X have been implicated in host cell shutoff, whereby IAV infection leads to a global degradation of cellular mRNAs in order to allow preferential translation of viral mRNAs by the cellular translation machinery. This strategy is commonly used by many viruses and additionally serves to reduce induction of immune related genes. *In vitro* studies using PA-X from the PR8 strain showed that PA-X is able to target host mRNAs for degradation^36,37^. However, PR8 PA-X has relatively low shutoff activity compared to PA-X proteins from avian IAV strains^13^. Here, we observed in an *in vivo* model of IAV infection that the lungs of mice infected with a PA-X-deficient PR8 virus contain comparable levels of host mRNAs at 48 h.p.i. with the exception of two genes encoding early type I IFNs, *Ifnb1* and *Ifna4*.

There are differences in the kinetics of induction among the genes encoding the multiple type I IFN subtypes. Phosphorylation of the constitutively expressed transcription factor IRF3 early in the cellular response to viral infection is a key event in the initial transcriptional activation of the mouse *Ifna4* and *Ifnb1* genes. IFN-α4 and IFN-β proteins are secreted by the infected cell and signal in an autocrine and paracrine manner by binding to the type I IFN receptor IFNAR on the surface of the same or a neighbouring cell. This triggers the activation of the intracellular JAK-STAT signalling pathway, in particular the rapid phosphorylation of STAT1 and STAT2 which, together with IRF9, form the Interferon-Stimulated Gene Factor 3 (ISGF3) complex. ISGF3 binds to interferon-stimulated response elements (ISREs) in the promoters of target genes including *Irf7* which, unlike *Irf3*, is not constitutively expressed. Subsequent activation of IRF7 protein by phosphorylation is required for the induction of the other *Ifna* genes, and therefore the amplification of the type I IFN response^15,16,38^. Our data suggest that PA-X acts to target the mRNAs of type I IFN genes induced early in infection by MAVS-dependent activation of IRF3, with no effect on the mRNAs of genes which depend on IFN signalling for their induction, such as *Ifna5/*6 and the ISGs *Ifi44*, *Ifit1* and *Isg20*. Transcriptional control of the *Il28a* gene, encoding IFN-λ2, has been proposed to be similar to that of the *Ifna* genes as opposed to *Ifnb1*^39,40^. This implies that PA-X may be able to selectively target *Ifnb1* and *Ifna4* mRNAs for degradation. However, it is unclear why increased mRNA levels do not correlate with increased protein levels as the mRNAs are polyadenylated; further investigation is required to address this question.

Mice are a widely used animal model for researching IAV infection. The infection of C57BL/6 mice with the lab adapted PR8 strain is one of the most commonly used models of IAV infection *in vivo*^14^. Although C57BL/6 mice lack functional Mx1, a major restriction factor for IAV^41^, the availability of mice lacking key components of the innate immune system on this genetic background provides a powerful means of dissecting the sensing of the virus by the host and the immune response induced. In this study, we used MAVS- and TLR7-deficient C57BL/6 mice to show that the effect of PA-X on *Ifnb1* and *Ifna4* mRNA accumulation is via a MAVS-dependent pathway. A number of earlier studies have used mice as an experimental model to assess the function of PA-X during IAV infection *in vivo*; however, there is no clear consensus regarding whether PA-X increases or decreases pathogenicity of influenza viruses in mice. Infection of mice with PA-X-deficient viruses of the 1918 H1N1^5^, 2009 pH1N1^9,10,42^ and H5N1^10–12^ strains suggested that PA-X has a role in reducing the pathogenicity of these viruses (reviewed by Hu *et al*.^7^). However, other studies using 2009 pH1N1^43^ and H9N2^44^ viruses found that PA-X increased the virulence of these strains in mice, suggesting that the contribution of PA-X to the pathogenicity of IAV viruses varies depending on the host and the virus strain. In some cases, increased levels of *Ifnb1* mRNA were detected in the lungs of mice infected with PA-X-deficient viruses; however, it is unclear whether this has a protective effect. Our data show that increased *Ifnb1* and *Ifna4* mRNA levels in the absence of PA-X are dependent on MAVS. The mechanism by which PA-X reduces MAVS-dependent *Ifnb1* and *Ifna4* expression, and the functional consequence of this, remains to be determined. In addition to degradation of *Ifnb1* and *Ifna4* mRNAs, direct interactions with RIG-I, MAVS or any of the other proteins involved in downstream signalling are possible, as described for NS1, PB2 and PB1-F2.

In summary, we show that the PA-X protein of the widely used experimental PR8 strain of IAV prevents accumulation of type I IFN mRNAs induced early in infection by a MAVS-dependent pathway. Understanding the role of PA-X in the virulence and pathogenicity of IAV should be a key objective of future studies.

## Methods

### Mice

All mice were on the C57BL/6 background. *Mavs*^−/−^ and *Tlr7*^−/−^ mice were a gift from C. Reis e Sousa and were originally from J. Tschopp^45^ and S. Akira^46^, respectively. This work was performed in accordance with the UK Animals (Scientific Procedures) Act 1986 and institutional guidelines for animal care. This work was approved by project licenses granted by the UK Home Office (PPL numbers 40/3583 and PC041D0AB) and was also approved by the Institutional Animal Ethics Committee Review Board at the University of Oxford.

### Cell culture

HEK293T (Open Biosystems), HEK293 (a gift from C. Reis e Sousa), p125-HEK^23^ and MDCK-SIAT1 cells (a gift from A. Townsend) were grown in DMEM (Sigma-Aldrich) supplemented with 10% heat-inactivated FCS and 2 mM L-glutamine. Cells were grown at 37 °C and 5% CO_2_. The absence of mycoplasma contamination was confirmed monthly for all cell lines. HEK293T were treated overnight with 100 U/ml of Universal type I IFN (IFN-A/D, Sigma) prior to viral infection. Sendai virus was purchased from ATCC (Cantrell strain).

### Production of a PA-X-deficient virus

PR8 WT and the PA-X-deficient PR8 virus PR8 FS were produced by reverse genetics as described previously^5,13^. Viruses were passaged in MDCK-SIAT cells. Experiments were performed using two independently propagated viral preparations.

### Viral growth curve

A549 cells were infected in serum-free medium at an MOI of 0.01 and after a 1 h absorption period at 37 °C, overlaid with media containing 0.14% BSA and 1 µg/ml L-(tosyl- amido-2-phenyl) ethyl chloromethyl ketone (TPCK)-treated trypsin (Worthington Biochemicals). Supernatants were harvested at varying times post infection and titrated by plaque assay on MDCK cells as previously described^47^.

### *In vivo* infections

C57BL/6, *Mavs*^−/−^ and *Tlr7*^−/−^ female mice were used at 6 to 10 weeks of age. Animals were age-matched within individual experiments. Mice were intranasally inoculated with 50 μl PR8 WT or PR8 FS diluted in DMEM or mock infected with DMEM only under light isofluorane (Isoflo®, Abbott Laboratories) anaesthesia. Animals were assessed for weight loss and signs of disease. Mice reaching 15% weight loss and/or exhibiting clinical signs of disease were euthanised.

### RNA extraction and qRT-PCR

Lungs were snap frozen in liquid nitrogen immediately after dissection and stored at −80 °C until being processed. Lungs were homogenised with glass beads (425–600 μm, Sigma-Aldrich) in TRIzol (Thermo Fisher Scientific) using a FastPrep F120 instrument (Thermo Savant). RNA was extracted using chloroform phase-separation following the manufacturer’s instructions and was further purified using RNeasy columns (Qiagen) including an on-column DNase I step. cDNA synthesis was performed with SuperScript II reverse transcriptase (Thermo Fisher Scientific) and random hexamer primers (Qiagen) or oligo(dT)_12–18_ primers (Thermo Fisher Scientific) as indicated. 30 ng of cDNA was amplified using Taqman Universal PCR Mix (Thermo Fisher Scientific) and Taqman probes (Applied Biosystems). Alternatively, 20 ng of cDNA was amplified using EXPRESS SYBR GreenER qPCR Supermix (Thermo Fisher Scientific) and DNA oligonucleotides (Sigma Aldrich). qPCR was performed on a 7800 real-time PCR system (Applied Biosystems). mRNA expression data were normalised to *Gapdh* and analysed by the comparative C_T_ method. The qPCR probes and primers used in this study are listed in Table 1.

**Table 1:** qPCR probes, oligonucleotides and antibodies used in this study.

qRT-PCR				
Taqman probes (Thermo Fisher Scientific)	Assay Probe ID	Oligos (Sigma Aldrich)	Sequence	Reference
Gene		Gene		
*Gapdh*	Cat. No. 4352932E	*M*	F: 5′-CTTCTAACCGAGGTCGAAACGTA	Shin *et al*. 2013, Virology Journal 10:303
*Ifnb1*	Mm00439546_s1		R: 5′-GGTGACAGGATTGGTCTTGTCTTTA	
*Ifna4*	Mm00833969_s1	*Gapdh*	F: 5′-CATGGCCTTCCGTGTTCCTA	Tsujita *et al*. 2006, PNAS 103:11946–11951
*Ifna5*	Mm00833976_s1		R: 5′-CCTGCTTCACCACCTTCTTGAT	
*Ifna6*	Mm01703458_s1			
*Ifi44*	Mm00505670_m1			
*Ifit1*	Mm00515153_m1			
*Isg20*	Mm00469585_m1			
*Il28a*	Mm04204158_gh			
*Ifng*	Mm01168134_m1			
**Oligos to generate probes for Northern blots**				
**Gene**	**Sequence**	**Template for PCR**	**Reference**	
*Ifnb1*	F: 5′-ATGAACAACAGGTGGATCC	pGEM-IFN-β	Schulz *et al*.^49^, Cell Host Microbe 7:354–361	
	R: 5′-GGCATCAACTGACAGGTCTT			
*Actb*	F: 5′-GACTTTGTACATTGTTTTG	cDNA from PR8 WT-infected mouse lung	This study	
	R: 5′-TGCACTTTTATTGGTCTCA			
**Oligos for ePAT**				
**Name**	**Sequence**	**Reference**		
PAT-anchor primer	5′-GCGAGCTCCGCGGCCGCGTTTTTTTTTTTT	Janicke *et al*.^19^, RNA 18:1289-1295		
TVN-PAT primer	5′-GCGAGCTCCGCGGCCGCGTTTTTTTTTTTTVN	Janicke *et al*.^19^, RNA 18:1289-1295		
mIfnb1 ePAT primer	5′-ACCTGTCAGTTGATGCCTCA	This study		
**Antibodies used for Western blot**				
**Antigen**	**Supplier**	**Host**	**Cat**. **number**	**Dilution**
PA	GeneTex	Rabbit pAb	GTX125932	1:1000
MAVS	ENZO Life Sciences	Rabbit pAb	ALX-210-929-C100	1:500
RIG-I	AdipoGen	Mouse mAb	AG-20B-0009	1:1000
p-IRF3	Cell Signaling Technology	Rabbit mAb	4947S	1:1000
β-actin	Sigma	Mouse mAb (HRP-coupled)	A3854	1:100,000

### TCID_50_ assays

Lungs were snap frozen in liquid nitrogen immediately after dissection and stored at −80 °C until being processed. Lungs were thawed on ice and homogenised with glass beads (425–600 μm, Sigma-Aldrich) in 1 ml viral growth medium (VGM; DMEM supplemented with 0.1% BSA, 10 mM HEPES, 2 mM L-glutamine, 100 U/ml penicillin and 0.1 mg/ml streptomycin) using a FastPrep F120 instrument (Thermo Savant). Homogenates were centrifuged at 16,000 × g for 3 min at 4 °C to pellet beads and debris. The virus-containing supernatant was diluted 1:250 in VGM and filtered using a 0.22 μm filter (Millipore). The filtrate was further diluted 1:30 in VGM to give the top dilution and a 1:3 dilution series of ten dilutions made. Determination of 50% tissue culture infective dose (TCID_50_) was performed using MDCK-SIAT1 cells, seeded 24 h earlier at a concentration of 3 × 10^4^ cells per well of a flat-bottomed 96 well plate. Cells were washed once in PBS before 100 μl of virus-containing supernatant was added, with each column containing 8 replicates of the same dilution. Two columns were used for positive (PR8 WT virus) and negative controls (VGM only). Following incubation at 37 °C for 1 h, 150 μl of TPCK-treated trypsin (Sigma Aldrich) was added to each well to give a final trypsin concentration of 1 μg/ml and the plates incubated for 72 h at 37 °C. The presence or absence of virus in each well was scored by incubating 50 μl of supernatant with 50 μl 1% (vol/vol) human red blood cells in a V-bottomed 96 well plate at room temperature for 1 h and tipping the plates to assess the presence or absence of virus-mediated haemagglutination. Wells were scored as positive or negative and TCID_50_ values calculated using the Reed and Muench method^48^.

### Western blot

Cells were lysed in 10 mM Tris HCl pH 7.5, 150 mM NaCl, 0.5 mM EDTA, 0.5% Igepal CA-630 (Sigma), 1 mM PMSF and protease inhibitor cocktail (Cell Signaling Technology) for 30 min on ice, pipetting every 10 min to mix. Protein lysates were cleared by centrifugation at 16,000 × g 4 °C for 10 min, boiled in Laemmli sample loading buffer (60 mM Tris HCl pH 6.8, 2% SDS, 10% glycerol, bromophenol blue, 5% beta-mercaptoethanol) for 5 min and run on Bis-Tris SDS-PAGE gels (NuPAGE, Thermo Fisher Scientific). Antibodies used for Western blot are listed in Table 1. Anti-rabbit and anti-mouse secondary antibodies coupled to HRP were from GE Healthcare.

### ELISA

Levels of type I IFNs in the BALf were quantified using a LumiKine Xpress mIFN-β ELISA (Invivogen) and a Lumikine mIFN-α ELISA (Invivogen) which detects multiple IFN-α subtypes. IP-10 was quantified using a IP-10/CXCL10 Matched Antibody Pair ELISA (eBioscience).

### Luciferase reporter assay

HEK cells stably expressing firefly luciferase under the control of the human *IFNB* promoter (p125-HEK cells) have been described previously^23^. Cells were seeded at 20,000 cells per well in a 96-well plate. The following day, cells were transfected with the indicated doses of RNA extracted from the lungs of mice complexed with 0.5 μl Lipofectamine 2000 (Thermo Fisher Scientific) per well. Activation of the *IFNB* promoter was assessed after 24 h using OneGlo luciferase assay reagent (Promega).

### Flow cytometry

Lungs were dissected and mechanically disrupted using scissors before incubation in RPMI containing 1 μg/ml type II collagenase (Worthington Biochemical Corporation) and 40 U/ml DNase I (Sigma Aldrich) at 37 °C for 60 min, resuspending after 30 min to facilitate tissue dissociation. Cells were filtered through a 70 μm cell strainer (BD Falcon), rinsing with RPMI before being pelleted at 400 × g for 5 min. The cell pellet was resuspended in 5 ml RBC lysis buffer (Qiagen), incubated at room temperature for 5 min then washed twice with 45 ml RPMI. Cells were resuspended in 1 ml PBS containing 10% (v/v) FCS and 2 mM EDTA (FACS buffer), passed over a 70 μm cell strainer and viable cells counted using a haemocytometer. 3 × 10^6^ cells of each sample were washed with PBS before incubating with LIVE/DEAD Fixable Aqua Dead Cell Stain (Invitrogen) diluted 1:200 in PBS for 30 min at room temperature. Cells were washed twice with FACS buffer and fixed with 4% PFA (v/v) in PBS for 15 min at room temperature before being permeabilised with 0.2% (w/v) saponin (Sigma Aldrich) in PBS containing 2% (w/v) BSA for 20 min at room temperature. Intracellular IAV NP protein was stained using an anti-NP-FITC monoclonal antibody (D67J, Thermo Fisher Scientific) diluted 1:40 in permeabilisation buffer for 30 min at room temperature followed by two washes in FACS buffer. Data were acquired on a BD LSR Fortessa X-20 (BD Biosciences) and analysed using FlowJo (v10.4.1).

### Northern blot

Northern blots were performed as previously described with some modifications^1,49^. Briefly, total RNA extracted from lungs was heat-denatured for 15 min at 75 °C before being electrophoresed on a 1.2% agarose gel containing 7% formaldehyde, alongside an RNA ladder (Life Technologies). Gels were run overnight at 4 °C in MOPS buffer (20 mM MOPS, 8 mM sodium acetate, 1 mM EDTA, pH 7.0). RNA was transferred by capillary transfer to a nylon membrane (Hybond N+, Amersham Biosciences) overnight after which the membrane was crosslinked with UV light (120 mJ/cm^2^) and hybridised with [α^32^P]-dCTP and [α^32^P]-dATP labelled probes diluted in hybridisation buffer (Ambion Ultrahyb) overnight at 42 °C. Primer sequences used to generate the probes are listed in Table 1.

### Extension Poly(A) Test (ePAT)

ePAT was performed according to Janicke *et al*.^19^ with some modifications. Briefly, 1 μl of 100 μM DNA oligonucleotide (‘PAT-anchor primer’) was annealed to 1 μg RNA extracted from IAV-infected lungs by incubating at 80 °C for 5 min then cooling to room temperature. A master mix containing 1 X SuperScript III buffer, 5 mM DTT, 0.5 mM dNTPs, 40 U RNaseOUT and 5 U of Klenow polymerase was added to give a final reaction volume of 20 μl, mixed and incubated at for 1 h at 37 °C, followed by 5 min at 80 °C to inactivate the polymerase and cooling to 55 °C for 1 min. The tubes were maintained at 55 °C while 200 U of SuperScript III Reverse Transcriptase (Invitrogen) was added. The samples were mixed and incubated for 1 h at 55 °C followed by 5 min at 80 °C to inactivate the reverse transcriptase. The resulting ePAT cDNA was diluted 1:6 with UltraPure distilled water (Invitrogen). A TVN-PAT cDNA control was generated by the same method using the TVN-PAT primer, which contains two 3′ variable bases, V and N, which lock the primer to the polyadenylation site during the reverse transcription step, which was performed at 42 °C instead of 37 °C. PCR amplification of the ePAT and TVN-PAT cDNA was performed in a 20 μl reaction containing 5 μl of cDNA, 1X AmpliTaq Gold 360 master mix (Applied Biosystems), 1 μM forward primer (‘mIfnb1 ePAT primer’) and 1 μM reverse primer (‘PAT-anchor primer’). Reactions were incubated at 94 °C for 10 min then 22–28 cycles of 94 °C for 20 sec, 60 °C for 20 sec, 72 °C for 20 sec and finally 1 min at 72 °C. PCR amplicons were visualised by loading 10 μl of each reaction on to a 2% high-resolution agarose gel (MetaPhor Agarose, Lonza) containing SYBR Safe (Invitrogen). Band intensity was calculated using Image Lab software (Bio-Rad Laboratories).

### Statistics

Statistical analysis was performed in GraphPad Prism v7.00 as detailed in the figure legends.

## Supplementary information

Supplementary Information

## Data Availability

All data generated or analysed during this study are included in this manuscript (and its Supplementary Information files).
