# Supplementary material for: PA-X antagonises MAVS-dependent accumulation of early type I interferon messenger RNAs during influenza A virus infection
Source: Sci Rep. 2019 May 10;9:7216. doi: 10.1038/s41598-019-43632-6 (PMC6510759; doi:10.1038/s41598-019-43632-6)
Supplement: Supplementary file 1 — Supplementary Information [file 41598_2019_43632_MOESM1_ESM.docx]

**Supplementary Information**

PA-X antagonises MAVS-dependent accumulation of early type I interferon messenger RNAs during influenza A virus infection

Rachel E. Rigby, Helen M. Wise, Nikki Smith, Paul Digard and Jan Rehwinkel

Supplementary Figure S1

Supplementary Figure S2

Supplementary Figure S3

Supplementary Figure S4

Supplementary Figure S5


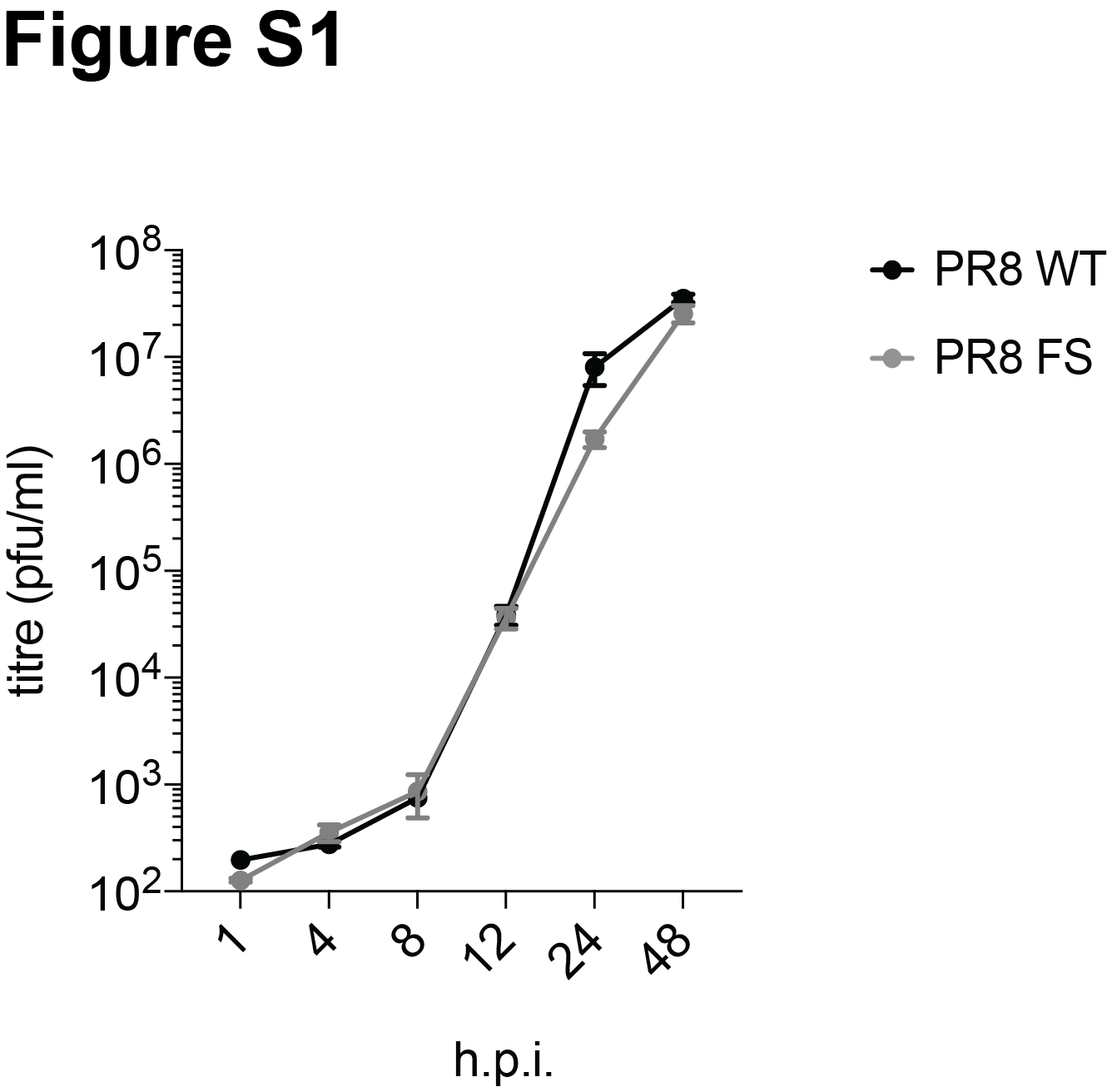


**Figure S1.** Growth curves of PR8 WT and PR8 FS viruses in A549 cells. A549 cells were infected at MOI 0.01 and supernatants titrated by plaque assay on MDCK cells at 1, 4, 8, 12, 24 and 48 h.p.i. Data are from one experiment + s.d. of technical duplicates.


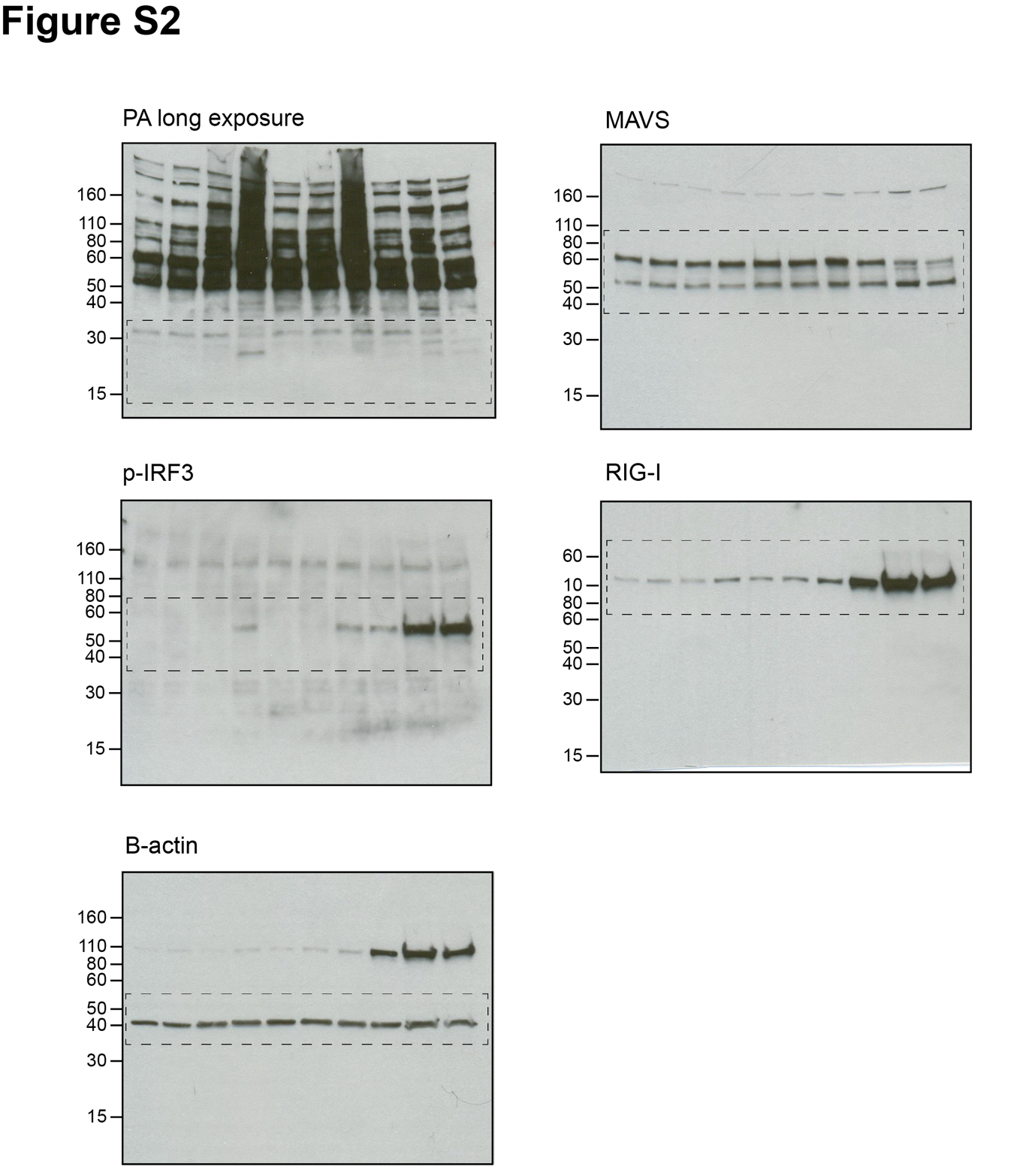


**Figure S2.** Full length Western blots from Fig. 1B. The regions shown in Fig. 1B are indicated by the dashed lines. The RIG-I blot was re-probed with antibody against B-actin.


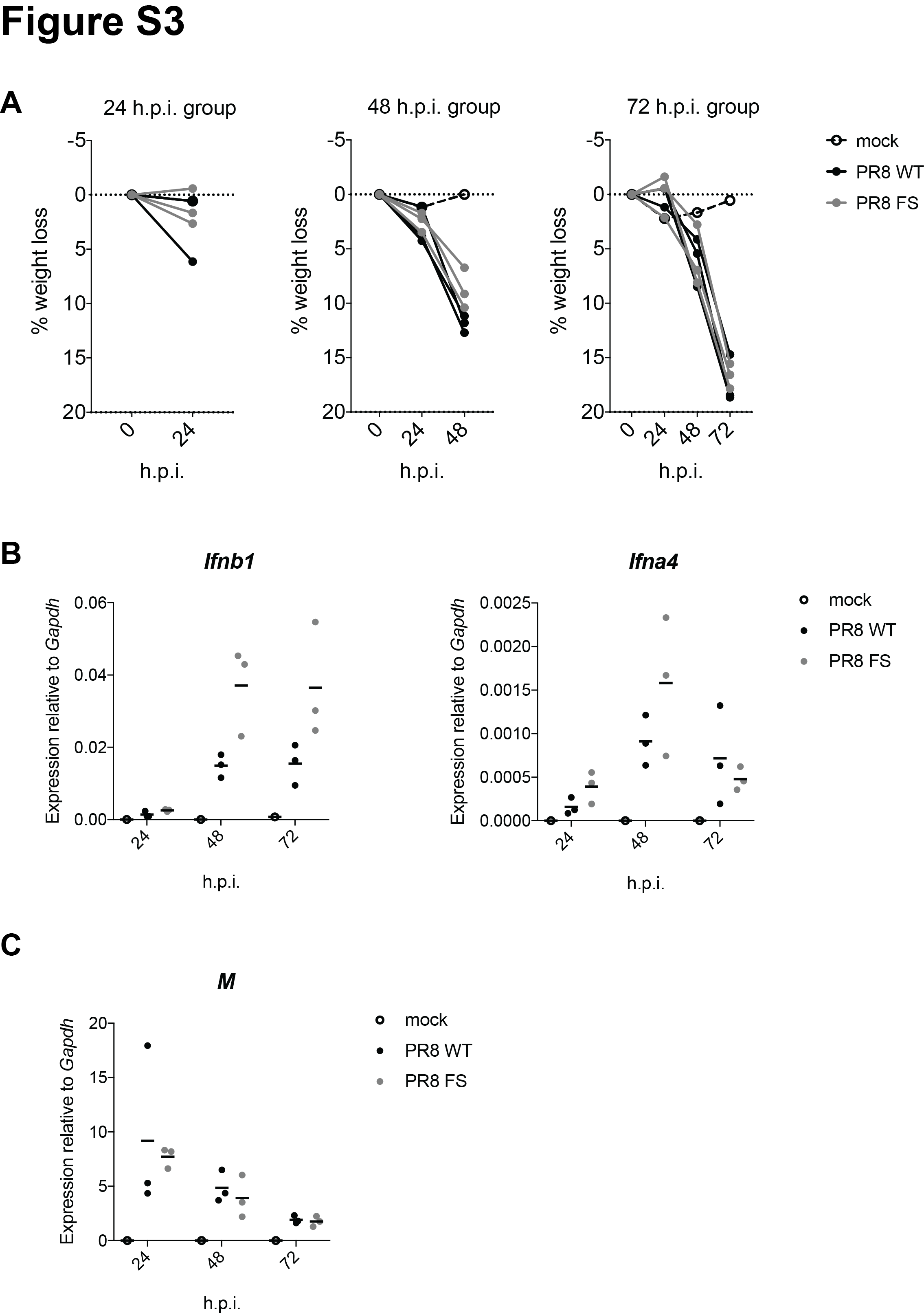


**Figure S3.** Timecourse of type I IFN gene expression following infection of wild-type mice with 50,000 pfu PR8 WT, PR8 FS or mock infection via the intranasal route. (A) Weight loss over the course of infection. (B) Levels of *Ifnb1* and *Ifna4* mRNA in the lung were measured by qRT-PCR. (C) Levels of the IAV M RNA were measured by qRT-PCR. Data shown are from 1 mouse (mock) and 3 mice per group (infected).


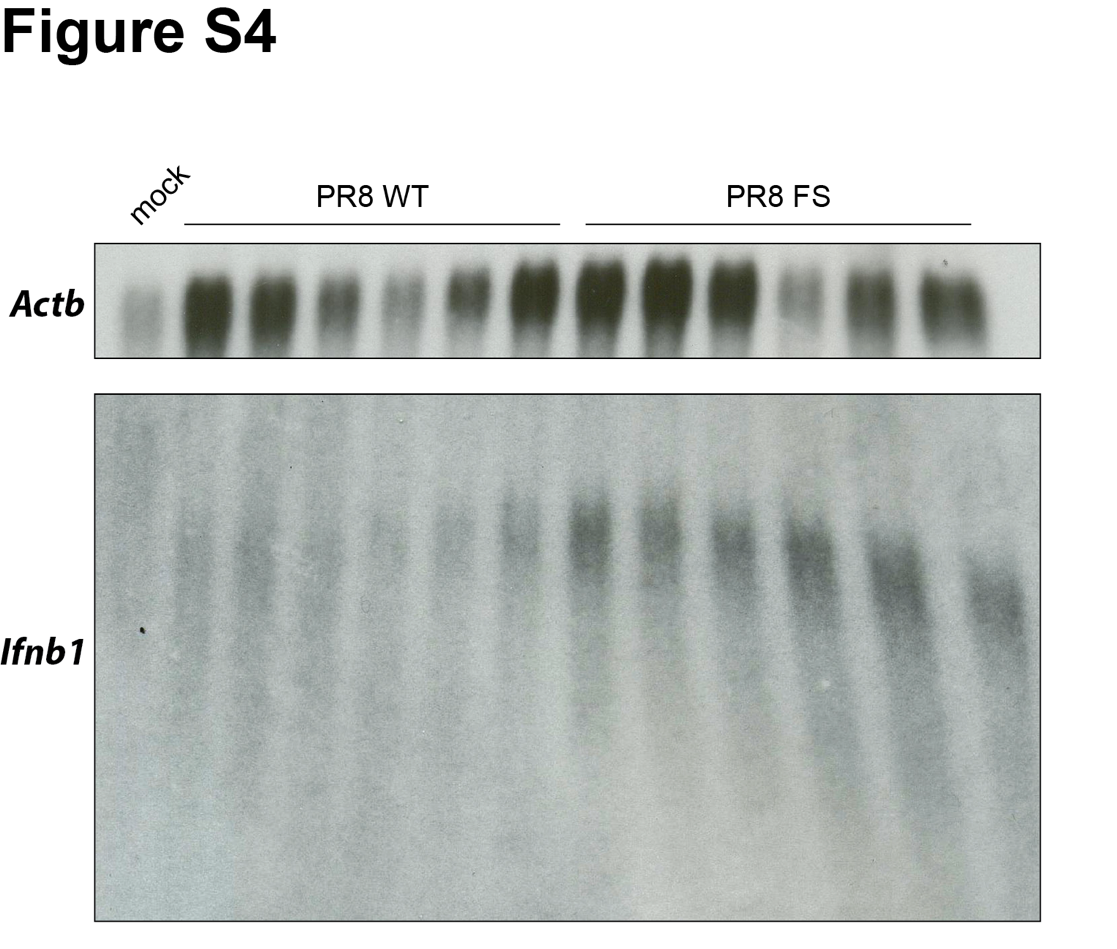


**Figure S4.** Northern blot for *Ifnb1* mRNA in the lungs of infected mice. Wild-type mice were infected with 50,000 pfu of PR8 WT or PR8 FS via the intranasal route. Lungs were harvested 48 h.p.i. and RNA extracted as described in the Methods section. 30 μg of total RNA for each sample was electrophoresed on a denaturing agarose gel and transferred to a nylon membrane. The membrane was probed with ^32^P-labelled probes for *Ifnb1* and *Actb*. Representative image of two independent experiments.

**
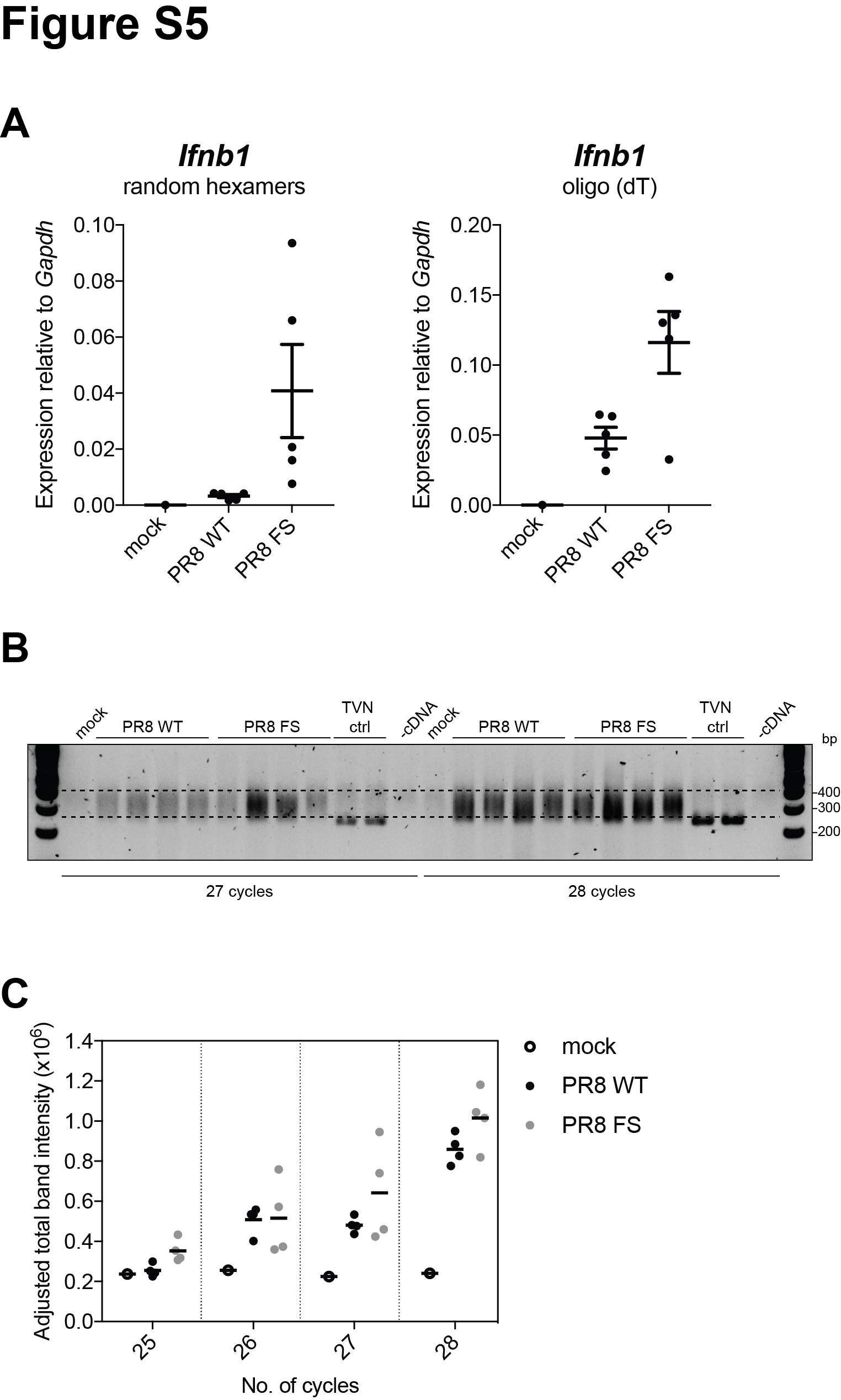
**

**Figure S5.** *Ifnb1* mRNA in the lungs of PR8 FS-infected mice is polyadenylated. RNA extracted from the lungs of wild-type mice infected with 50,000 pfu PR8 WT or PR8 FS for 48 h.p.i. was extracted as described in the Methods section. (A) *Ifnb1* expression was analysed in the same samples by qRT-PCR using random hexamer or oligo(dT) primers to generate the cDNA. Data are represented as *Ifnb1* expression relative to the housekeeping gene *Gapdh*, + s.e.m. (B, C) Semi-quantitative measurement of *Ifnb1* mRNA poly(A) tail length in total RNA from the lungs of infected mice using extension Poly(A) Test (ePAT). Briefly, the 3' end of adenylated RNA was extended from an ePAT DNA oligonucleotide template annealed via an oligo (dT) stretch using Klenow polymerase before being reverse transcribed. The resulting cDNA was used as a template for PCR amplification using a gene-specific primer and a universal reverse primer for the indicated number of cycles. A TVN-PAT cDNA control was generated using a primer identical to the ePAT primer with the addition of two 3' variable bases V and N, which lock the primer to the polyadenylation site during the reverse transcription step. PCR amplification of the TVN control results in a product corresponding to the size of the amplicon with a fixed (A_12_) poly(A) tail. (B) The length of the poly(A) tail present on endogenous mRNA can be calculated by comparing the size of the ePAT PCR products to the TVN control. (C) Quantification of band intensity from ePAT PCR reactions using 25, 26, 27 or 28 cycles (latter two shown in (B)). Band intensities were calculated using Image Lab software (Bio-Rad Laboratories) and are represented as band intensity adjusted for background subtraction.
